# Genetic variation related to caffeine metabolism or response during exercise

**DOI:** 10.1186/1550-2783-12-S1-P53

**Published:** 2015-09-21

**Authors:** Nanci S Guest, Joseph Jamnik, Christopher Womack, Ahmed El-Sohemy

**Affiliations:** 1 Faculty of Medicine, Department of Nutritional Sciences, University of Toronto, Toronto, ON, M5S 3E2, Canada; 2 Department of Kinesiology, James Madison University, Harrisonburg, VA 22807, USA

## Background

Caffeine use for improved athletic performance has variable effects. Caffeine can exert a wide variety of physiologic effects that range from adverse (e.g., anxiety, increased heart rate, nervousness) to pleasurable (e.g., alertness, elevated mood, increased energy), which could be associated with individual genetic differences.

## Methods

We examined whether a panel of 25 SNPs in 19 genes that might be related to caffeine metabolism or response modified exercise performance, or were associated with any physiologic outcomes during exercise. Subjects were trained male cyclists (n = 33) who underwent a double-blind placebo-controlled crossover trial to test the effects of caffeine (6 mg/kg) on various performance parameters during a computer-simulated 40 km time trial. The 25 SNPs were genotyped using the Sequenom MassARRAY^® ^system, and caffeine-genotype interactions on time trial time, VO_2 _max, heart rate, respiratory exchange ratio and rate of perceived exertion were assessed using repeated measures analysis of variance.

## Results

There was a significant interaction between caffeine and rs4410790, a SNP located near the aryl hydrocarbon receptor (*AHR*) gene, on heart rate during the time trial (p = 0.007). Compared with placebo, caffeine supplementation increased heart rate (HR) to a greater extent in carriers of the T allele (n = 19; placebo = 155 ± 12 bpm; caffeine = 165 ± 11 bpm p = < 0.0001) compared with CC homozygotes (n = 14; placebo = 164 ± 15 bpm; caffeine 167 ± 14 bpm p = 0.11).

## Conclusion

Our findings show that a polymorphism near the *AHR *gene was associated with a greater elevation in HR during a 40-kilometer time trial after caffeine ingestion, but had no effect on performance.

